# Antineoplastic Activity of New Transition Metal Complexes of 6-Methylpyridine-2-carbaldehyde-N(4)-ethylthiosemicarbazone: X-Ray Crystal Structures of [VO_2_(mpETSC)] and [Pt(mpETSC)Cl]

**DOI:** 10.1155/2010/149149

**Published:** 2010-07-01

**Authors:** Shadia A. Elsayed, Ahmed M. El-Hendawy, Sahar I. Mostafa, Bertrand J. Jean-Claude, Margarita Todorova, Ian S. Butler

**Affiliations:** ^1^Department of Chemistry, McGill University, Montreal, QC, Canada H3A 2K6; ^2^Chemistry Department, Faculty of Science, Mansoura University, Damietta 34517, Egypt; ^3^Chemistry Department, Faculty of Science, Mansoura University, Mansoura, Egypt; ^4^Department of Medicine, Royal Victoria Hospital, Montreal, QC, Canada H3A 1A1

## Abstract

New complexes of dioxovanadium(V), zinc(II), ruthenium(II), palladium(II), and platinum(II) with 6-methylpyridine-2-carbaldehyde-N(4)-ethylthiosemicarbazone (HmpETSC) have been synthesized. The composition of these complexes is discussed on the basis of elemental analyses, IR, Raman, NMR (^1^H, ^13^C, and ^31^P), and electronic spectral data. The X-ray crystal structures of [VO_2_(mpETSC)] and [Pt(mpETSC)Cl] are also reported. The HmpETSC and its [Zn(HmpETSC)Cl_2_] and [Pd(mpETSC)Cl] complexes exhibit antineoplastic activity against colon cancer human cell lines (HCT 116).

## 1. Introduction

**Figure 1:**
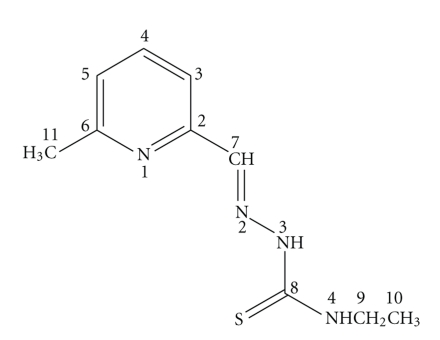
Structure of 6-methylpyridine-2-carbaldehyde-N(4)-ethylthiosemicarbazone (HmpETSC).

Interest in thiosemicarbazone chemistry has flourished for many years, largely as a result of its wide range of uses, for example, as antibacterial, antifungal, chemotherapeutic, and bioanalytical agents [1–6]. One particular area of thiosemicarbazone chemistry that has been increasing in importance recently involves biologically active metal complexes of thiosemicarbazone-based chelating (NNS) agents. As the coordination of the metal ions to thiosemicarbazones improves their efficacy and improve their bioactivity [6]. In this concept, zinc(II), palladium(II), and platinum(II) complexes of pyridine-2-carboxaldehyde thiosemicarbazone and substituted pyridine thiosemicarbazone were tested against human cancer breast and bladder cell lines and found to be selectively cytotoxic to these malignant cell carcinoma [7, 8]. We have previously studied the chemotherapeutic potential of a series of Mo(VI), Pd(II), Pt(II), and Ag(I) complexes with N,O; N,S and O,O-donors. These complexes were found to display significant anticancer activity against* Ehrlich ascites tumor cell* (EAC) in albino mice [9–12]. Copper(II) complexes of 6-methylpyridine-2-carbaldehyde and its N(4)-methyl, ethyl, and phenyl thiosemicarbazones have been reported as well as their activity against pathogenic fungi [13]. In this paper, we report the synthesis and spectroscopic characterizations of new complexes of 6-methylpyridine-2-carbaldehyde-N(4)-ethylthiosemicarbazone (HmpETSC, Figure 1) with V(V), Zn(II), Ru(II), Pd(II), and Pt(II). The X-ray crystal structures of [VO_2_(mpETSC)] and [Pt(mpETSC)Cl] have been reported. Also, the anticancer activity of HmpETSC and its Zn(II) and Pd(II) complexes toward colon cancer human cell lines has been tested.

## 2. Experimental

All reagents were purchased from Alfa/Aesar and Aldrich. [RuCl_2_(PPh_3_)_3_] was prepared as previously reported in [14]. Infrared spectra were recorded using a Nicolet 6700 Diamond ATR spectrometer in the 200–4000 cm^−1^ range. Raman spectra were recorded on in Via Renishaw spectrometer using 785 nm laser excitation. NMR spectra were recorded on Varian Mercury 500 MHz spectrometer in DMSO-d6 with TMS as reference. Electronic spectra were recorded in DMF using Hewlett-Packard 8453 Spectrophotometer. Elemental analyses and X-ray crystallography were performed in Université De Montréal. The human cancer cell lines were obtained from the American Type Culture Collection (ATCC catalog number): HCT116 human colorectal carcinoma (CCL-247). Cells were maintained in Roswell Park Memorial Institute (RPMI-1640) medium (Wisent Inc., St-Bruno, Canada) supplemented with 10% FBS, 10 mM HEPES, 2 mM L-gutamine, and 100 *μ*g/mL penicillin/streptomycin (GibcoBRL, Gaithersburg, MD). All assay cells were plated 24 hours before drug treatment.

### 2.1. Preparation of the Ligand: 6-Methylpyridine-2-carboxaldehyde-N(4)-ethylthiosemicarbazone (HmpETSC)

6-Methylpyridine-2-carboxaldehyde (1.21 g, 10 mmol) in ethanol (10 cm^3^) was added to N(4)-ethylthiosemicarbazide (1.19 g, 10 mmol) in ethanol-water solution (V/V 1 : 1, 80 cm^3^) followed by the addition of drops of glacial acetic acid. The reaction mixture was refluxed for 3 hours. The precipitate obtained was filtered off, washed with water and ethanol, and recrystallized from ethanol then dried in vacuo. m. p. = 201°C. Elemental analytical calculation for C_10_H_13_N_4_S: C, 54.0, H, 6.4; N, 25.2; S, 14.4% found C, 54.0, H, 6.3; N, 25.1; S, 14.2%.

### 2.2. Preparation of the Complexes

#### 2.2.1. [VO_2_(mpETSC)]

To a solution of HmpETSC (0.044 g, 0.2 mmol) in acetonitrile (10 cm^3^), [VO(acac)_2_] (0.053 g, 0.2 mmol) was added. The reaction mixture was refluxed for 1 hour. Upon cooling the yellowish green solution, orange precipitate was obtained. It was filtered off, washed with ethanol, and dried in vacuo. The brown crystals suitable for X-Ray crystallography were obtained by a slow evaporation of a solution of the complex in acetonitrile. The yield was 50% (based on the metal). Elemental analytical calculation for C_10_H_13_N_4_O_2_SV: C, 39.5; H, 4.3; N, 18.4; S, 10.5% found C, 39.4; H, 4.0; N, 18.2; S, 10.3%. 

#### 2.2.2. [Zn(HmpETSC)Cl_2_]

A methanolic solution (10 cm^3^) of HmpETSC (0.044 g, 0.2 mmol) was added to ZnCl_2_ (0.027 g, 0.2 mmol) in methanol (10 cm^3^). The reaction mixture was refluxed for 2 hours, and the off-white product obtained was filtered off, washed with methanol, then dried in air. The yield was 35% (based on the metal). Elemental analytical calculation for C_10_H_14_Cl_2_N_4_SZn: C, 33.5; H, 3.9; N, 15.6; S, 8.9% found C, 33.7; H, 3.7; N, 15.5; S, 8.8%.

#### 2.2.3. [Ru(PPh_3_)_2_(mpETSC)_2_]

A hot ethanolic solution of HmpETSC (0.044 g, 0.2 mmol) was added to [RuCl_2_(PPh_3_)_3_] (0.1 g, 0.1 mmol). Et_3_N (0.02 cm^3^, 0.2 mmol) was then added and the reaction mixture was refluxed for 2 hours. The red brown solution was filtered and upon reducing the volume by evaporation a brown solid was isolated. It was filtered off, washed with ethanol and ether. The yield was 33% (based on the metal). Elemental analytical calculation for C_56_H_56_N_8_P_2_RuS_2_: C, 63.0; H, 5.3; N, 10.5; S, 6.0% found that C, 62.8; H, 5.1; N, 10.4; S, 5.8%.

#### 2.2.4. [Pd(mpETSC)Cl]

A solution of K_2_[PdCl_2_] (0.1 g, 0.3 mmol) in water (2 cm^3^) was added to HmpETSC (0.066 g, 0.3 mmol) in methanolic solution of KOH (0.018 g, 0.3 mmol; 15 cm^3^). The reaction mixture was stirred at room temperature for 24 hours. The orange precipitate was filtered off, washed with water methanol, and finally air-dried. Yield was 60% (based on metal). Elemental analytical calculation for C_10_H_13_ClN_4_PdS: C, 33.1; H, 3.6; N, 15.4; S, 8.8% found C, 33.4; H, 3.2; N, 15.2; S, 8.5%.

#### 2.2.5. [Pt(mpETSC)Cl]

An aqueous solution (3 cm^3^) of K_2_PtCl_4_ (0.042 g, 0.1 mmol) was added dropwise to a methanolic solution of HmpETSC (0.022 g, 0.1 mmol; 15 cm^3^). The reaction mixture was stirred overnight at room temperature. Upon evaporation of the solvent, fine red crystals were observed. These were suitable for single crystal X-ray crystallography. Yield was 25% (based on metal). Elemental analytical calculation for C_10_H_13_ClN_4_PtS: C, 26.6; H, 2.9; N, 12.4; S, 7.1% found C, 26.8; H, 2.8; N, 12.1; S, 6.9%.

### 2.3. X-Ray Crystallography

The crystal structure were measured on The X-Ray Crystal Structure Unit, using a Bruker Platform diffractometer, equipped with a Bruker MART 4 K Charger-Coupled Device (CCD) Area Detector using the program APEX II and a Nonius Fr591 rotating anode (Copper radiation) equipped with Montel 200 optics. The crystal-to-detector distance was 5 cm, and the data collection was carried out in 512 × 512 pixel mode. The initial unit cell parameters were determined by the least-squares fit of the angular setting of strong reflections, collected by a 10.0 degree scan in 33 frames over three different parts of the reciprocal space (99 frames total). One complete sphere of data was collected. 

The crystals of [VO_2_(mpETSC)] and [Pt(mpETSC)Cl] were mounted on the diffractometer, and the unit cell dimensions and intensity data were measured at 200 K. The structures were solved by the least-squares fit of the angular setting of strong reflections based on *F^2^*. The relevant crystal data and experimental conditions along with the final parameters are reported in Table 1. 

### 2.4. Antineoplastic Testing

In the growth inhibition assay, HCT116 cells were plated at 5,000 cells/well in 96-well flat-bottomed microtiter plates (Costar, Corning, NY). After 24-hour incubation, cells were exposed to different concentrations of each compound continuously for four days. Briefly, following HmpETSC and its Zn(II) and Pd(II) complexes treatment, cells were fixed using 50 *μ*l of cold trichloroacetic acid (50%) for 60 minutes at 4°C, washed with water, stained with 0.4% sulforhodamine B (SRB) for 4 hours at room temperature, rinsed with 1% acetic acid, and allowed to dry overnight [15]. The resulting colored residue was dissolved in 200 *μ*l Tris base (10 mM, pH 10.0), and optical density was recorded at 490 nm using a microplate reader ELx808 (BioTek Instruments). The results were analyzed by Graph Pad Prism (Graph Pad Software, Inc., San Diego, CA), and the sigmoidal dose response curve was used to determine 50% cell growth inhibitory concentration (IC_50_). Each point represents the average of two independent experiments performed in triplicate.

## 3. Results and Discussion

### 3.1. Synthesis and Physical Properties of the Complexes

The preparative reactions for the complexes can be represented by the following equations:

$$\begin{array}{cc} & \text{VO}{\left(\text{acac}\right)}_{2}+\text{HmpETSC}\overset{{\text{CH}}_{3}\text{CN},\text{T}}{\to }\left[{\text{VO}}_{2}\left(\text{mpETSC}\right)\right]\\ & {\text{ZnCl}}_{2}+\text{HmpETSC}\overset{\text{MeOH},\text{T}}{\to }\left[\text{Zn}\left(\text{HmpETSC}\right){\text{Cl}}_{2}\right]\\ & \left[\text{Ru}{\left({\text{PPh}}_{3}\right)}_{3}{\text{Cl}}_{2}\right]+\text{HmpETSC}\overset{\text{EtOH}/{\text{Et}}_{3}\text{N},\text{T}}{\to }\\ & \;\;\;\;\;\;\;\;\;\;\;\;\left[\text{Ru}{\left({\text{PPh}}_{3}\right)}_{2}{\left(\text{mpETSC}\right)}_{2}\right]\\ & {\text{K}}_{2}{\text{PdCl}}_{4}+\text{HmpETSC}\overset{{\text{H}}_{2}\text{O}/\text{MeOH},\text{T}}{\to }\left[\text{Pd}\left(\text{mpETSC}\right)\text{Cl}\right]\\ & {\text{K}}_{2}{\text{PtCl}}_{4}+\text{HmpETSC}\overset{{\text{H}}_{2}\text{O}/\text{MeOH},\text{T}}{\to }\left[\text{Pt}\left(\text{mpETSC}\right)\text{Cl}\right] \end{array}$$

All the complexes are microcrystalline or amorphous powder, stable in the normal laboratory atmosphere, and slightly soluble in common organic solvent but completely soluble in DMF and DMSO.

### 3.2. Infrared and Raman Spectra

The infrared and Raman spectral assignments of the ligand, HmpETSC, and its reported complexes are listed in Table 2. HmpETSC has the characteristic thioamide moiety (-HN-C(S)NHEt), which can be present in either thione or thiol form (Figure 1) [16, 17]. The IR and Raman spectra of HmpETSC show the absence of absorption band in 2500–2600 cm^−1^ region indicating the presence of the free HmpETSC in thione form [18]. HmpETSC shows a strong IR band at 1589 cm^−1^, observed at 1607 cm^−1^ in the Raman, which is corresponding to the azomethine, *v*(HC=N), group [13, 19]. In the spectra of the complexes, the shift of this band to higher frequency is observed, suggesting the participation of azomethine nitrogen in the coordination to metal ions [20, 21]. This feature is further supported by the shift of *v*(N-N) band in the free ligand (at 992 and 1006 cm^−1^ in IR and Raman, respectively) to higher frequencies upon complexation [18, 22]. On the other hand, the participation of the deprotonated thiol sulfur in coordination was indicated by the shift of the IR band at 812 cm^−1^ (at 824cm^−1^ in the Raman) in the free ligand to lower frequencies in the complexes [19, 23]. This view is supported by the absence of *v*(N(3)H) vibration with the observation of new band near 1570 cm^−1^ in the complexes which may assign to *v*(N(3)=C) [24]. Furthermore, the coordination of pyridine nitrogen atom is indicated through the positive shift of the ring deformation band in HmpETSC near 582 and 586 cm^−1^ in the IR and Raman spectra, respectively [25]. Both IR and Raman spectral data suggest mononegative tridentate (N, N, S^−^) behavior of mpETSC^−^. In case of [Zn(HmpETSC)Cl_2_], the *v*(N(3)H) band is observed at lower wave number as the thione sulfur participates in coordination [26]. Also, there is no shift observed in the pyridine ring deformation mode, that is, HmpETSC acts as a neutral bidentate ligand through both thione sulfur and azomethine nitrogen atoms [25].

 The spectra of the complexes show that new bands in the IR and Raman near 450 cm^−1^may assign to *v*(M-N) [27]. Also, the far IR and Raman spectra show new bands near 325 and 300 cm^−1^ can be assigned to *v*(M-S) and *v*(M-Cl), respectively [9, 10].

In the 940–920 cm^−1^ region the IR spectrum of the complex [VO_2_(mpETSC)] shows two strong bands characteristic of the *cis*-VO_2_ moiety [28, 29]. 

The presence of the coordinated PPh_3_ in the complex [Ru(PPh_3_)_2_(mpETSC)_2_] is confirmed by the appearance of the characteristic *v*(P-C_ph_) and *δ*(C-CH) band at 1085 and 720 cm^−1^, respectively [30].

### 3.3. NMR Spectra

Table 3 shows the ^1^H-NMR spectral data of HmpETSC and its reported complexes in DMSO-d_6_ (see Figure 1 for numbering scheme) which are in a great agreement with those reported in the literature [13, 31, 32]. In the spectrum of free HmpETSC, the singlet observed at *δ* 11.62 ppm assigned to N(3)H is disappeared in the spectra of the complexes indicating that the coordination takes place through the deprotonated thiol sulfur atom [33]. In [Zn(HmpETSC)Cl_2_], this band is observed at *δ* 11.63 ppm, confirming the data observed in the IR and Raman spectra that the coordination of HmpETSC to Zn(II) occurs through the thione sulfur atom [34]. As expected. the singlet observed at *δ* 8.02 ppm in the free ligand assigned to the azomethine H(7)C=N proton shows downfield shift in the complexes (*δ* 8.22–8.71 ppm), due to the involvement of azomethine nitrogen in coordination [16, 33]. The spectrum of HmpETSC shows singlet at *δ* 8.66 ppm assigned to the thioamide N(4)H proton, this signal is shifted upfield upon complexation [32, 34]. This feature may be due to the sequence of establishment of hydrogen bonds formation [35, 36]. The spectrum of HmpETSC exhibits triplet and quartiplet signals at *δ*1.14 and 3.58 ppm assigned to H(10) and H(9), respectively. Also, the pyridine protons appear in *δ* 7.22–8.059 ppm region [33]. As expected, these protons are shifted downfield complexes (except in case of [Zn(HmpETSC)Cl_2_]) due to the decrease in the electron density caused by electron withdrawal by the metal ions from the sulfur, azomethine nitrogen, and pyridine nitrogen atoms. 

^13^C-NMR assignments of the HmpETSC and its complexes are listed in Table 4 and are in agreement with the reported data [13]. The spectrum of the free ligand shows number of resonances at *δ* 14.98, 24.49, 38.81, 117.69, 123.78, 137.14, 142.74, 153.18, 158.28, and 177.28 ppm, assigned to C(10), C(11), C(9), C(5), C(3), C(4), C(7), C(6), C(2), and C(8), respectively. In the complexes, the resonances of the carbon atoms adjacent to the coordination sites (C(7), C(8), C(2), and C(6)) are shifted downfield relatively to their positions in the free ligand [37, 38]. This feature may be due to an increase in current brought about by coordination to azomethine nitrogen, pyridine nitrogen, and deprotonated thiol sulfur atoms [25, 39]. In the spectrum of [Zn(HmpETSC)Cl_2_] complex, the resonances arising from C(6), C(2) are more or less in the same positions as in the free ligand indicating that HmpETSC acts as a neutral bidentate ligand through thione sulfur and azomethine nitrogen atoms [25]. 

The ^31^P-NMR spectrum of [Ru(PPh_3_)_2_(mpETSC)_2_] shows a sharp singlet at *δ* 52.48 ppm, suggesting the presence of the two PPh_3_ groups in *trans*-configuration [30].

### 3.4. Electronic Spectra

The electronic spectrum of HmpETSC shows bands at 340 and 300 nm assigned to *π* → π* and *n* → π* of the azomethine and pyridine ring transitions, respectively [40, 41]. In the complexes, both transitions undergo blue shifts indicating the coordination *via* the azomethine and pyridine nitrogen atoms [42].

The electronic spectra of [M(mpETSC)Cl] (M(II) = Pd, Pt) show that two bands near 475 and 330 nm can be assigned to ^1^A_1g_→ ^1^B_1g_ and ^1^A_1g_ → ^1^E_g_ transitions, respectively, in square planar configurations [9–12]. 

The electronic spectrum of the diamagnetic [Ru^II^(PPh_3_)_2_(mpETSC)_2_] shows bands at 532, 354, and 393 nm (^1^A_1g_ → ^1^T_1g_, ^1^A_1g_ → ^1^T_2g_, and ligand (p-dp) transitions, respectively). These are attributed to a low-spin octahedral geometry around Ru(II) [10–12].

The electronic spectrum of the diamagnetic [VO_2_(mpETSC)] shows that two bands at 440 and 360 nm may be assigned to MLCT and *n*-π* transitions, respectively [43]. 

### 3.5. X-Ray Crystallography

**Figure 2:**
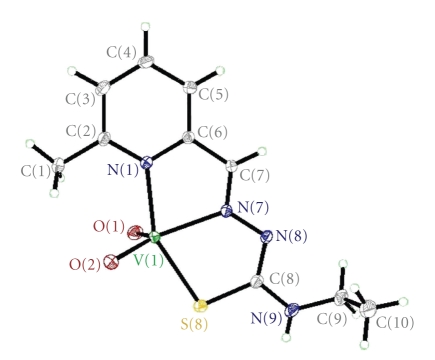
Structure of [VO_2_(mpETSC)] with numbering scheme.

**Figure 3:**
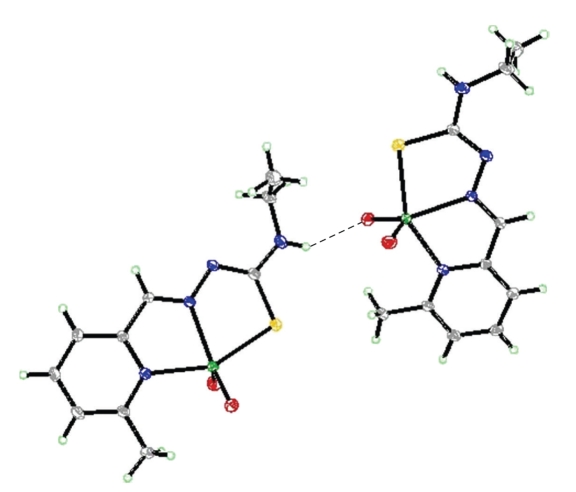
Hydrogen bonding interaction in the lattice of [VO_2_(mpETSC)].

**Figure 4:**
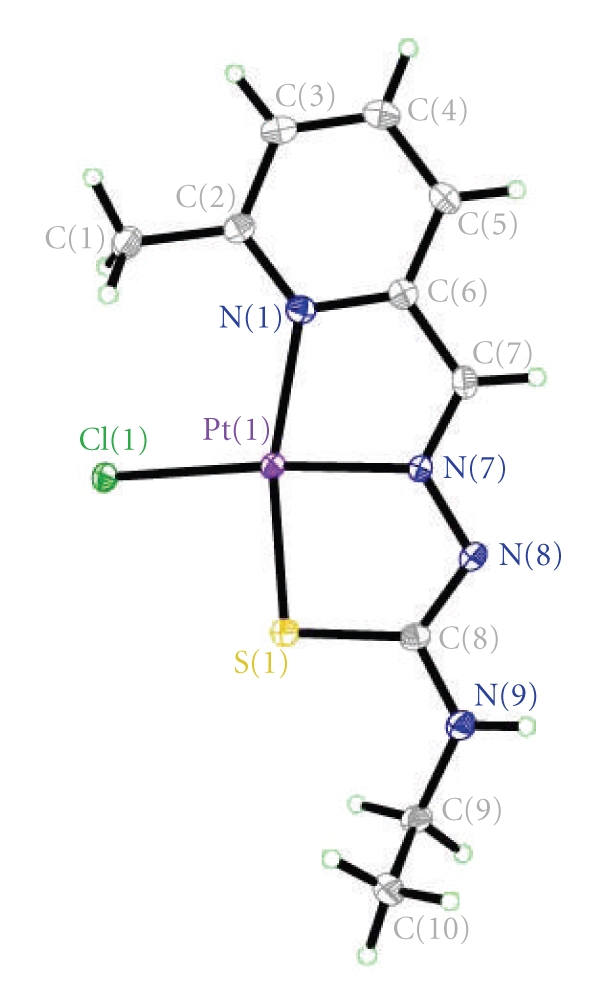
Structure of [Pt(mpETSC)Cl] with numbering scheme.

**Figure 5:**
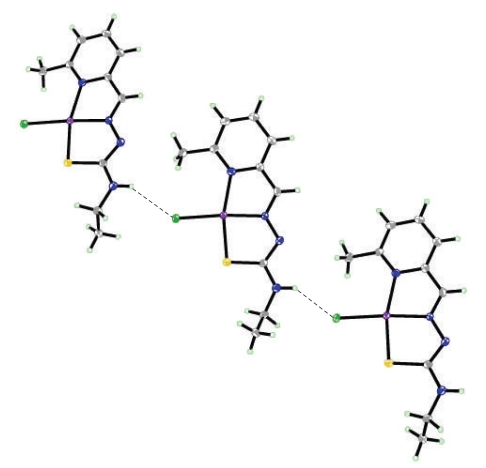
Hydrogen bonding interaction in the lattice of [Pt(mpETSC)Cl].

The structure of the complexes [VO_2_(mpETSC)] and [Pt(mpETSC)Cl], together with the atoms numbering scheme adopted is shown in Figures 2, 3, 4, and 5, respectively. The selected bond distances and bond angles of the complexes are listed in Tables 5, 6, 7, and 8, respectively. The complexes [VO_2_(mpETSC)] and [Pt(mpETSC)Cl] are crystallized in monoclinic lattice with space group symmetry P21/c and P21/n, respectively. 

The X-ray crystal structure of [VO_2_(mpETSC)] shows that the vanadium(V) atom has a distorted square pyramidal environment in which mpETSC^−^ is coordinated to the metal ion as a tridentate chelating agent binding *via* the deprotonated thiolat sulfur S(8), the azomethine nitrogen N(7), and pyridine nitrogen N(1) atoms, yielding two five-membered chelate rings (Figure 2) with bond distances (V-N(1), 2.1333(14) Å, V-N(7), 2.1651(13) Å, and V-S(8), 2.3800(5) Å). The other two sites are occupied by oxo ligands O(1) and O(2) in *cis*-configuration. The O(1) occupies the basal position with mpETSC^−^ donor while the O(2) occupies the apical position (V-O(1), 1.6145(12) Å and V-O(2), 1.6356(12) Å) [42]. In the present complex [VO_2_(mpETSC)], the bond distances C(8)-N(8), 1.322(2) Å and C(7)-N(7), 1.287(2) Å are not intermediate between single and double bonds, but they are closer to double bonds. Also, the N(7)-N(8), 1.322(2) Å bond length is very close to a single bond (Table 5). Moreover, the C(8)-S(8) bond length in the complex (1.7472(7) Å) is intermediate between a C-S double bond (1.62 Å) and a C-S single bond (1.82 Å), indicating that this bond maintains a partial double-bond character [42]. The bond angles data, N(1)-V-N(7), 75.37(5)  °; N(7)-V-S(8), 76.48(4)°, O(2)-V-S(8), 96.73(5)°, O(1)-V-O(2), 107.64(7)°, O(1)-V-N(1), 96.08(6)°, indicate that the complex has a distorted square pyramidal geometry, which may be attributed to the restricted bite angles of mpETSC^−^ [44, 45]. The network structure is stabilized by the intermolecular hydrogen bonding interaction, N(9)H…….O(2) bond (Table 6, Figure 3).

In case of [Pt(mpETSC)Cl], mpETSC^−^ is also coordinated platinum(II) in the same tridentate manner, and chloride atom has taken up the fourth coordination site on Pt(II) in planar configuration (Figure 4). The bond lengths, Pt-N(1), 2.116(8) Å, Pt-N(7), 1.979(5) Å, Pt-S(1), 2.2533(8) Å, Pt-Cl(1), 2.3178(3) Å, in the complex are longer than those found in other reported square-planar platinum(II) complexes with N,S-donors [34–36, 42]. The data show that [Pt(mpETSC)Cl] has short N-N and long C-S bond lengths (Table 7) compared with other reported complexes. The bond angles of N(1)-Pt-S(1), 165.40(8)° and N(7)-Pt-Cl(1), 174.13(12)° are deviated substantially from that expected for a regular square-planar geometry. The monomer units of this complex are linked together into polymeric net chain through N(9)H…..Cl intermolecular hydrogen bonds as shown in Table 8 and Figure 5 [46].

### 3.6. Antineoplastic Activity

**Figure 6:**
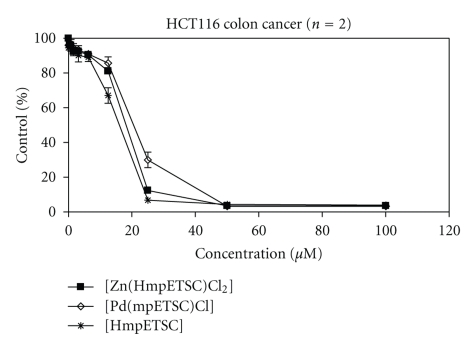
Antineoplastic activity in human colon carcinoma HCT116 cells by a growth inhibition SRB assay after 96-hour treatment of HmpETSC, [Zn(HmpETSC)Cl_2_], and [Pd(mpETSC)Cl].

HmpETSC, [Zn(HmpETSC)Cl_2_], and [Pd(mpETSC)Cl] were tested for their antineoplastic activity against the human colon tumor cell lines (HCT 116). The three compounds exhibited remarkable growth inhibitory activities with mean IC_50_ values of 14.59, 16.96, and 20.65 *μ*M, respectively (Table 9 and Figure 6). 2-Formy and 2-acetylpyridine-N(4)-ethylthiosemicarbazones and their complexes [M(f4Et)_2_] and [M(Ac4Et)_2_] (M(II) = Pd, Pt, f4Et, Ac4Et = 2-formy and 2-acetylpyridine-N(4)-ethylthiosemicarbazone) have been tested in a panel of human colon, breast, and ovary tumor cell lines and were found to exhibit very remarkable growth inhibitory activities with mean IC_50 _values of 0.9–0.5 nM [47]. It is clear that the complexation of f4Et and Ac4Et in [Pd(f4Et)_2_], [Pd(Ac4Et)_2_], [Pt(f4Et)_2_], and [Pt(Ac4Et)_2_] modified their activities towards the tumor cells [47]. The complex [Zn(HmpETSC)Cl_2_] exhibits much better antineoplastic activity against HCT 116 compared to [Pd(mpETSC)Cl] which is more active than [Pt(mpETSC)Cl]. The substitution and modes of chelations of HmpETSC in the complexes [Zn(HmpETSC)Cl_2_] and [Pd(mpETSC)Cl] are different than both f4Et and Ac4Et in the reported Pd(II) and Pt(II) complexes [48]. As reported, *cis*-N_2_ and *cis*-S_2_ configuration in the complexes [M(f4Et)_2_] and [M(Ac4Et)_2_] (M(II) = Pd, Pt) display their significant antitumor activity [46, 49]. Also, in the [Zn(HmpETSC)Cl_2_], HmpETSC acts as a neutral bidentate chelating agent which is different than its behavior (mononegative tridentate) in [Pd(mpETSC)Cl]. Furthermore, the presence of the intermolecular hydrogen bonds in the later complex may reduce its antineoplastic activity [48]. 

## 4. Conclusion

The aim of this report is to study the structure and antineoplastic activity of 6-methylpyridine-2-carbaldehyde-N(4)-ethylthiosemicarbazone (HmpETSC) and its complexes with dioxovanadium(V), zinc(II), ruthenium(II), palladium(II), and platinum(II). The X-ray crystal structure of the complexes [VO_2_(mpETSC)] and [Pt(mpETSC)Cl] was reported. HmpETSC behaves as mononegative tridentate through the pyridine nitrogen, azomethine nitrogen and the deprotonated thiol sulfur atoms except in case of Zn(II) complex, it behaves as a neutral bidentate through azomethine nitrogen and thione sulfur atoms. HmpETSC and its Zn(II) and Pd(II) complexes show antineoplastic activity against the human colon tumor cell lines (HCT 116).

## Figures and Tables

**Table 1:** Crystal data and structure refinement for VO_2_(mpETSC) and Pt(mpETSC)Cl.

	[VO_2_(mpETSC)]	[Pt(mpETSC)Cl]
Empirical formula	C_10_H_13_N_4_ O_2_SV	C_10_H_13_ClN_4_PtS
Formula weight	304.24	451.84
Temperature	200 K	150 K
Wavelength	1.54178 Å	1.54178 Å
Crystal system	Monoclinic	Monoclinic
Space group	P21/c	P21/n
Unit cell dimensions		
a(Å), *α* (°)	8.5583(2), 90^o^	12.9824(2), 90
b(Å), *β* (°)	13.4934(3), 03.679(1)^o^	b = 7.0655(1). 94.454(1)^0^
c(Å), *γ* (°)	11.2697(3), 90^o^	c = 13.6601(2), 90
Volume (Å^3^)	1264.52(5) (Å^3^)	1249.22(3) (Å^3^)
Z, Density (calculated) g/cm^3^	4; 1.598 g/cm^3^	4; 2.402 g/cm^3^
Absorption coefficient	8.122 mm^−1^	24.402 mm^−1^
F(000)	624	848
Crystal size	0.26 × 0.10 × 0.06 mm	0.12 × 0.08 × 0.02 mm
Theta range for data collection (°)	5.20 to 72.30°	4.53 to 72.13
Index ranges	−10 ≤ *h* ≤ 10, −16 ≤ *h* ≤16, −13 ≤ *l* ≤ 13	−15 ≤ *h* ≤ 15, −8 ≤ *k* ≤ 8, −16 ≤ *l* ≤ 16
Reflections collected	16371	15858
Independent reflections	2468 [R_int _ = 0.033]	2442 [R_int _ = 0.045]
Absorption correction	Semi-empirical from equivalents	Semi-empirical from equivalents
Max. and min. transmission	0.6143 and 0.3013	0.6138 and 0.3359
Refinement method	Full-matrix least-squares on F^2^	Full-matrix least-squares on F^2^
Data/restraints/parameters	2468/0/169	2442/0/157
Goodness-of-fit on F^2^	1.150	1.065
Final R indices [I>2sigma(I)]	R_1_ = 0.0318, wR_2_ = 0.0881	R_1_ = 0.0277, wR_2_ = 0.0951
R indices (all data)	R_1_ = 0.0326, wR_2_ = 0.0887	R_1_ = 0.0307, wR_2_ = 0.0993
Extinction coefficient		0.00036(6)
Largest diff. peak and hole	0.414 and −0.711 e/Å^3^	1.579 and −1.242 e/Å^3^

**Table 2:** Infrared and Raman spectral data of HmpETSC and its complexes^*a*^.

Compound	*v*(NH)	*v*(HC=N)	*v*(C=C)	*v*(N=CS)	*v*(N–N)	*v*(CS)	*v*(M– N)	*v*(M–S)	*v*(M–Cl)
HmpETSC	3267	1589	1530	—	992	812	—	—	—
		**1607**	**1579**		**1006**	**824**			
[VO_2_(mpETSC)]	3214	1652	1613	1576	1017	787	427		926^b^
		** 1651**	**1570**	**1586**	**1019**	**754**	**427**	**343**	937^b^
[Zn(HmpETSC)Cl_2_]	3290	1625	1596		1009	805	466		
		**1626**	**1598**		**1009**	**793**	**427**	**317**	**300**
[Ru(PPh_3_)_2_(mpETSC)_2_]	3383	1572	1528	1479	999	788	465		
[Pd(mpETSC)Cl]	3286	1608	1582	1572	1008	784	454		
		**1617**	**1580**	**1570**	**1022**	**787**	**462**	**345**	**297**
[Pt(mpETSC)Cl]	3322	1607	1580	1570sh	1020	779	424		
		**1609**	**1584**	**1564**	**1009**	**779**	**421**	**330**	**306**

^a^Raman data are in *bolds*,^b^ *v*(O=V=O) sym and asym.

**Table 3:** ^1^H-NMR spectral data of HmpETSC and its complexes.

Compound	H(3) (d)	H(4) (t)	H(5) (d)	H(7)CH=N (s)	H(9) (q)	H(10) (t)	Me(py) (s)	N(3)H (s)	N(4)H (s)
HmpETSC	8.06	7.71	7.22	8.02	3.58	1.14	2.45	11.62	8.67
[VO_2_(mpETSC)]	7.56	8.11	7.67	8.58	3.32	1.12	2.48	—	8.19
[Zn(HmpETSC)Cl_2_]	8.02	7.73	7.23	8.71	3.58	1.13	2.46	11.63	8.67
[Ru(PPh_3_)_2_(mpETSC)_2_]	7.55	7.45	7.38	8.63	3.34	0.88	2.38	—	—^a^
[Pd(mpETSC)Cl]	7.55	7.95	7.38	8.22	3.23	1.07	2.49	—	7.95
[Pt(mpETSC)Cl]	7.55	8.55	7.46	8.22	3.31	1.08	2.48	—	7.98

^a^ Overlapped with Ph protons.

**Table 4:** ^13^C-NMR spectral data of HmpETSC and its complexes.

Compound	C(2)	C(3)	C(4)	C(5)	C(6)	C(HC=N)	(C(C=S))	C(9)	C(10)	C(11)
HmpETSC	158.28	123.78	137.14	117.69	153.18	142.74	177.28	38.81	14.98	24.49
[VO_2_(mpETSC)]	163.16	127.39	142.76	123.26	153.75	149.43	175.46	39.82	14.85	26.34
[Zn(HmpETSC)Cl_2_]	158.01	124.01	137.59	118.06	152.82	142.22	177.25	38.83	14.94	24.07
[Ru(PPh_3_)_2_(mpETSC)_2_]	157.32	127.08	137.82	117.45	155.44	143.41	183.48	36.37	15.94	24.94
[Pd(mpETSC)Cl]	163.54	127.87	140.56	123.52	157.64	149.90	178.56	41.85	14.74	25.70
[Pt(mpETSC)Cl]	164.02	129.06	140.61	123.56	157.88	146.54	180.45	40.55	14.92	25.93

**Table 5:** Selected bond lengths and bond angles for [VO_2_(mpETSC)].

bond lengths (Å)		Bond angles (°)	
V(1)–O(1)	1.6145(12)	O(2)–V(1)–S(8)	96.73(5)
V(1)–O(2)	1.6356(12)	N(1)–V(1)–S(8)	151.43(4)
V(1)–N(1)	2.1333(14)	N(7)–V(1)–S(8)	76.48(4)
V(1)–N(7)	2.1651(13)	C(8)–S(8)–V(1)	100.39(6)
V(1)–S(8)	2.3800(5)	C(2)–N(1)–C(6)	118.72(14)
S(8)–C(8)	1.7472(17)	C(2)–N(1)–V(1)	125.52(11)
N(1)–C(2)	1.351(2)	C(6)–N(1)–V(1)	115.75(11)
N(1)–C(6)	1.361(2)	C(7)–N(7)–N(8)	116.94(13)
N(7)–C(7)	1.287(2)	C(7)–N(7)–V(1)	116.04(10)
N(7)–N(8)	1.3708(17)	N(8)–N(7)–V(1)	127.01(10)
N(8)–C(8)	1.322(2)	C(8)–N(8)–N(7)	111.43(13)
N(9)–C(8)	1.339(2)	C(8)–N(9)–C(9)	124.07(16)
N(9)–C(9)	1.454(2)	N(1)–C(2)–C(3)	120.41(16)
C(1)–C(2)	1.494(2)	N(1)–C(2)–C(1)	119.13(15)
C(2)–C(3)	1.398(2)	C(3)–C(2)–C(1)	120.45(15)
C(3)–C(4)	1.379(3)	C(4)–C(3)–C(2)	120.71(15)
C(4)–C(5)	1.390(2)	C(3)–C(4)–C(5)	118.81(16)
C(5)–C(6)	1.385(2)	C(6)–C(5)–C(4)	118.35(16)
C(6)–C(7)	1.451(2)	N(1)–C(6)–C(5)	122.94(16)
C(9)–C(10)	1.509(3)	N(1)–C(6)–C(7)	115.08(14)
		C(5)–C(6)–C(7)	121.98(15)
		N(7)–C(7)–C(6)	117.71(14)
		N(8)–C(8)–N(9)	118.62(15)
		N(8)–C(8)–S(8)	124.50(12)
		N(9)–C(8)–S(8)	116.87(13)
		N(9)–C(9)–C(10)	112.49(17)
		O(1)–V(1)–O(2)	107.64(7)
		O(1)–V(1)–N(1)	96.08(6)
		O(2)–V(1)–N(1)	101.30(6)
		O(1)–V(1)–N(7)	113.29(6)
		O(2)–V(1)–N(7)	139.07(6)
		N(1)–V(1)–N(7)	75.37(5)
		O(1)–V(1)–S(8)	99.35(5)

**Table 6:** Bond lengths [Å] and angles [°] related to the hydrogen bonding for [VO_2_(mpETSC)].

D-H	..A	d(D-H)	d(H..A)	d(D..A)	<DHA
N(9)–H(9)	O(2) no. 1	0.82(2)	2.30(2)	2.994(2)	144(2)

Symmetry transformations used to generate equivalent atoms: no. 1 −*x* + 1, and *y* − 1/2, −*z* + 3/2.

**Table 7:** Selected bond lengths and bond angles for the [Pt(mpETSC)Cl] complex.

bond lengths (Å)		Bond angles (°)	
Pt(1)–N(7)	1.979(5)	C(8)–S(1)–Pt1	95.02(11)
Pt(1)–N(1)	2.116(3)	C(2)–N(1)–C(6)	118.6(3)
Pt(1)–S(1)	2.2533(8)	C(2)–N(1)–Pt1	132.4(2)
Pt(1)–Cl(1)	2.3178(15)	C(6)–N(1)–Pt1	109.0(2)
S(1)–C(8)	1.757(3)	C(7)–N(7)–N(8)	121.8(5)
N(1)–C(2)	1.350(4)	C(7)–N(7)–Pt1	116.1(4)
N(1)–C(6)	1.370(5)	N(8)–N(7)–Pt1	121.9(3)
N(7)–C(7)	1.287(8)	C(8)–N(8)–N(7)	113.4(4)
N(7)–N(8)	1.365(6)	C(8)–N(9)–C(9)	127.1(3)
N(8)–C(8)	1.333(5)	N(1)–C(2)–C(3)	120.3(3)
N(9)–C(8)	1.331(4)	N(1)–C(2)–C(1)	119.7(3)
N(9)–C(9)	1.449(4)	C(3)–C(2)–C(1)	120.0(3)
C(1)–C(2)	1.494(4)	C(4)–C(3)–C(2)	121.1(3)
C(2)–C(3)	1.400(5)	C(3)–C(4)–C(5)	118.4(3)
C(3)–C(4)	1.370(5)	C(6)–C(5)–C(4)	119.1(3)
C(4)–C(5)	1.389(5)	N(1)–C(6)–C(5)	122.3(3)
C(5)–C(6)	1.381(5)	N(1)–C(6)–C(7)	116.5(4)
C(6)–C(7)	1.426(8)	C(5)–C(6)–C(7)	121.2(4)
C(9)–C(10)	1.491(5)	N(7)–C(7)–C(6)	117.7(6)
		N(9)–C(8)–N(8)	116.8(3)
		N(9)–C(8)–S(1)	118.8(3)
		N(8)–C(8)–S(1)	124.4(3)
		N(9)–C(9)–C(10)	113.1(3)
		N(7)–Pt1–N(1)	80.15(16)
		N(7)–Pt1–S(1)	85.25(14)
		N(1)–Pt1–S(1)	165.40(8)
		N(7)–Pt1–Cl1	174.13(12)
		N(1)–Pt1–Cl1	105.02(8)
		S(1)–Pt1–Cl1	89.57(4)

**Table 8:** Bond lengths (Å) and angles (°) related to the hydrogen bonding for [Pt(mpETSC)Cl].

D-H	..A	d(D-H)	d(H..A)	d(D..A)	<DHA
N(9)–H(9)	CL1 no. 1	0.88	2.62	3.372(3)	143.6

Symmetry transformations used to generate equivalent atoms: no. 1 *x* + 1/2, −*y* + 3/2, and *z* + 1/2.

**Table 9:** Antineoplastic activity in human colon tumor cell lines (HCT116) by growth inhibition SRB assay after 96-hour treatment.

Compound	HmpETSC	[Zn(HmpETSC)Cl_2_]	[Pd(mpETSC)Cl]
IC_50_, *μ*M	14.59	16.96	20.65
SD	0.81	0.46	1.60
